# Molecular epidemiology of childhood neuronal ceroid-lipofuscinosis in Italy

**DOI:** 10.1186/1750-1172-8-19

**Published:** 2013-02-02

**Authors:** Filippo Maria Santorelli, Barbara Garavaglia, Francesco Cardona, Nardo Nardocci, Bernardo Dalla Bernardina, Stefano Sartori, Agnese Suppiej, Enrico Bertini, Dianela Claps, Roberta Battini, Roberta Biancheri, Mirella Filocamo, Francesco Pezzini, Alessandro Simonati

**Affiliations:** 1 IRCCS Stella Maris-Molecular Medicine Unit, Pisa, Italy; 2 “C. Besta”-Molecular Neurogenetics and Child Neurology and Psychiatry Units, IRCCS Foundation Neurological Institute, Milan, Italy; 3 Department of Pediatric and Child Neurology and Psychiatry-Pediatric Neurology Unit, University of Rome, Rome, Italy; 4 Department of Life and Reproduction Sciences-Section of Child Neurology and Psychiatry, University of Verona, Verona, Italy; 5 Department of Pediatrics-Pediatric Neurology Unit, University of Padua, Padua, Italy; 6 IRCCS Pediatric Hospital Bambino Gesù-Molecular Medicine and Child Neurology Units, Rome, Italy; 7 IRCCS Istituto G Gaslini-"UO Neuropsichiatria Infantile" and "Centro di diagnostica genetica e biochimica delle malattie metaboliche", Genoa, Italy; 8 Department of Neurological, Neuropsychological, Morphological, Motor Sciences-Sections of Neurology (Child Neurology and Psychiatry) and Neuropathology, University of Verona, Piazzale LA Scuro 1, Verona, 37134, Italy

**Keywords:** Childhood NCL, NCL Genes, Epidemiology, Italy

## Abstract

**Background:**

To review the descriptive epidemiological data on neuronal ceroid lipofuscinoses (NCLs) in Italy, identify the spectrum of mutations in the causative genes, and analyze possible genotype-phenotype relations.

**Methods:**

A cohort of NCL patients was recruited through CLNet, a nationwide network of child neurology units. Diagnosis was based on clinical and pathological criteria following ultrastructural investigation of peripheral tissues. Molecular confirmation was obtained during the diagnostic procedure or, when possible, retrospectively.

**Results:**

One hundred eighty-three NCL patients from 156 families were recruited between 1966 and 2010; 124 of these patients (from 88 families) were tested for known NCL genes, with 9.7% of the patients in this sample having not a genetic diagnosis. Late infantile onset NCL (LINCL) accounted for 75.8% of molecularly confirmed cases, the most frequent form being secondary to mutations in *CLN2* (23.5%). Juvenile onset NCL patients accounted for 17.7% of this cohort, a smaller proportion than found in other European countries. Gene mutations predicted severe protein alterations in 65.5% of the CLN2 and 78.6% of the CLN7 cases. An incidence rate of 0.98/100,000 live births was found in 69 NCL patients born between 1992 and 2004, predicting 5 new cases a year. Prevalence was 1.2/1,000,000.

**Conclusions:**

Descriptive epidemiology data indicate a lower incidence of NCLs in Italy as compared to other European countries. A relatively high number of private mutations affecting all NCL genes might explain the genetic heterogeneity. Specific gene mutations were associated with severe clinical courses in selected NCL forms only.

## Background

The neuronal ceroid lipofuscinoses (NCLs) are the most common group of inherited, progressive neurodegenerative diseases of childhood, which are secondary to abnormal intralysosomal storage of autofluorescent material and show specific ultrastructural features. In the vast majority of patients, onset occurs in the pre-school years and is characterized by a combination of visual impairment, cerebellar ataxia, and signs and symptoms of diffuse telencephalic involvement, such as seizures, behavioral disturbances, and cognitive deterioration. The disease leads to progressive degeneration of cortical and cerebellar structures with secondary fiber tract atrophy. The outcome is always fatal; death occurs within a few years of the disease onset, in early adulthood, or even during the third or fourth decade of life, depending on the phenotype [1]. A clinical diagnosis is supported by ultrastructural evidence of abnormally shaped cytosomes in peripheral blood or skin biopsy [2].

The NCLs are inherited as autosomal recessive traits, although a rare, autosomal dominant adult-onset form has been identified [3]. The disease shows broad clinical and allelic heterogeneity; age at onset and, more recently, genetic characterizations have led to the development of a new molecular classification and novel diagnostic algorithms [4,5]. To date, fewer than 400 mutations in 12 human genes have been entered in the NCL database [6]. Nonetheless, approximately 10% of all patients do not have any of the known genetic forms of NCL and further heterogeneity is thus anticipated.

Information on the actual incidence and prevalence of childhood NCL is based mainly on data gathered from clinical and morphological studies conducted in the pre-genetic era [7-13]. Over the past 10 years, routine use of molecular genetics to corroborate clinical diagnoses has made it possible to obtain more accurate epidemiological data on the NCLs, which have been shown to have a worldwide distribution [14]. Moreover, some studies seem to indicate a higher-than-expected incidence in specific geographical regions [15,16]. In this study, we investigated the frequency of childhood-onset NCLs in Italy, examining clinical and molecular data collected mainly through a collaborative network (CLNet) of paediatric neurology units applying common diagnostic protocols.

## Patients and methods

The study included a large cohort of children born between 1966 and 2010. The data on all but five of these children were collected through CLNet; the remaining five were patients investigated by institutions not belonging to the network, but whose clinical data were available from the literature and from medical records [17,18] (S. Berkovic, personal communication). Diagnosis of NCL was based on clinical evaluation, neurophysiological investigations (EEG, multiple evoked potentials), neuroradiological findings, biochemical assays (when available), and ultrastructural analysis of blood lymphocytes and/or skin biopsies. Blood samples were obtained for DNA analysis in about 75% of the cohort. Molecular investigations of the *CLN1-3*, *CLN5-8* and *CLN10* genes in peripheral blood DNA and the rapid detection of the common 1.02Kb deletion of *CLN3* were performed using previously described procedures [19-25]. *ATP13A2* and *KCTD7* (recently reported to be mutated in CLN12 [26] and CLN14 [27], respectively) were not analyzed.

Descriptive epidemiological studies were conducted with reference to a 13-year period: 1992–2004. Demographic data for incidence and prevalence analyses were obtained from the Italian National Institute of Statistics (http://www.demo.istat.it). Cumulative NCL incidence was evaluated as described elsewhere [7]. Genotype data were used for molecular analysis as well as for genotype-phenotype correlations. The type and location of specific mutations were compared to the disease phenotypes as defined in the CLNet clinical database to establish their severity and identify possible population-specific variants. In addition, we correlated clinical diagnoses in Italian patients with frequency and severity of individual mutations.

Investigations were performed following signed, informed consent by childrens’ parents (or caregivers) according to the rules of the local ethical committees of each CLNet Unit.

## Results

### Descriptive epidemiology

One hundred eighty-three patients with childhood-onset NCL, born between 1966 and 2010 and belonging to 156 families were identified in the CLNet database. The sample comprised 81 boys, 66 girls, and 36 patients whose gender could not be ascertained because the access codes were not available. The investigated cohort includes patients reported in an earlier study [8]. General study population data are summarized in Table 1. There emerged no characteristic pattern of distribution of the patients with NCLs across the country, and the small geographical clusters that did emerge were related to familial cases. Thirty children (16.4%) were not of Italian ancestry, being either born in Italy to foreign parents, or adopted from abroad. Fourteen of these children originated from the Balkans, three from Eastern Europe, nine from Middle Eastern countries, and one each from Saharan Africa, India, Sri Lanka, and Nepal.

**Table 1:** Analytical data of the cohort of patients according to clinical NCL classification

**NCL Form**	**Patients (# cases;%)**	**Families**	**Molecular Investigations**
**INCL**	**10 (5.5)**	**7**	**8**
LINCL*	**124 (67.7)**	**109**	**94**
JNCL**	**49 (26.8)**	**40**	**22**
***Total***	**183**	**156**	**124**

* including so-termed variant (*) and protracted forms (**).

According to the clinical diagnosis of the all cohort (including patients whose diagnosis was not ascertained molecularly, but by neurophysiological data and ultrastructural findings), Late Infantile NCL (LINCL) was the most frequent subtype, affecting 67.7% of the patients. Infantile NCL (INCL) and Juvenile NCL (JNCL, including the so-called protracted form) accounted for 5.5% and 26.8%, respectively (see Table 1).

One hundred twenty-four patients were tested for mutations in the known NCL genes, and a molecular diagnosis was obtained in 112 of them (90.3%); the other twelve cases remained undiagnosed (Table 2). The clinical and ultrastructural findings of the 59 patients not undergoing DNA analyses were consistent with: INCL (n=2), LINCL (n=29), and JNCL (n=28).

**Table 2:** Molecular findings in 124 NCL patients according to both clinical form and mutated gene

**GENE**	**INCL**	**LINCL**	**JNCL**	**Total (%)**
***CLN1***	**6**	**9**	**2**	**17 (13.7)**
***CLN2***	**0**	**29**	**0**	**29 (23.5)**
***CLN3***	**0**	**0**	**16**	**16 (12.9)**
***CLN5***	**0**	**7**	**0**	**7 (5.6)**
***CLN6***	**0**	**18**	**3**	**21 (16.9)**
***CLN7***	**0**	**14**	**0**	**14 (11.3)**
***CLN8***	**0**	**7**	**0**	**7 (5.6)**
***CLN10***	**0**	**1**	**0**	**1 (0.8)**
***CLNX***	**2**	**9**	**1**	**12 (9.7)**
**Total (%)**	**8 (6.5)**	**94 (75.8)**	**22 (17.7)**	**124**

The mutations found in this cohort were scattered among all the examined NCL genes, none of which appeared to be overrepresented. *CLN2* mutations accounted for about 24% of the cases, all showing the LINCL phenotype. Mutations in *CLN6* were present in 21 patients (16.9%), affected by different phenotypes, including LINCL (18 cases) and JNCL (3 cases). Lower mutation rates were observed for *CLN1*, *CLN3* and *CLN7*. Three phenotypes were observed in association with mutations in *CLN1*, the LINCL one being the most common [28]. In accordance with figures published in a pre-DNA study [8], the number of CLN3-mutated patients was relatively low, accounting for 12.9% of the molecularly investigated cases. Even lower frequencies of *CLN5*, *CLN8* and *CLN10* mutations were observed. *CLN8* was the only gene not associated with familial cases, even though instances of parental consanguinity were rare and only a few of the kindred had multiple affected sibs.

Twelve cases, whose clinical and ultrastructural features were consistent with a diagnosis of NCL, remained undiagnosed, even after screening of NCL genes. Eight of them, from three families, were not of Italian origin (two from the Balkans and one from Sri Lanka).

For the purpose of conducting an incidence analysis, the 69 patients (from 59 families; 38 boys and 31 girls) born between 1992 and 2004 were matched with the total number of live births recorded in the same period in Italy: this gave an overall NCL incidence, in Italy, of 0.98/100,000 (that is, 1 in 102,041), predicting five new cases a year. A genetic diagnosis was obtained in 66 of these children (95.7%): 48 harboured mutations in genes associated with LINCL, which thus accounted for 69.6% of the cases. Only six patients with mutations in *CLN3* were born during the incidence study period (corresponding to 8.7% of this group), a figure which is in accordance with the low frequency of *CLN3* mutations in Italy (see Table 2).

**Figure 1:**
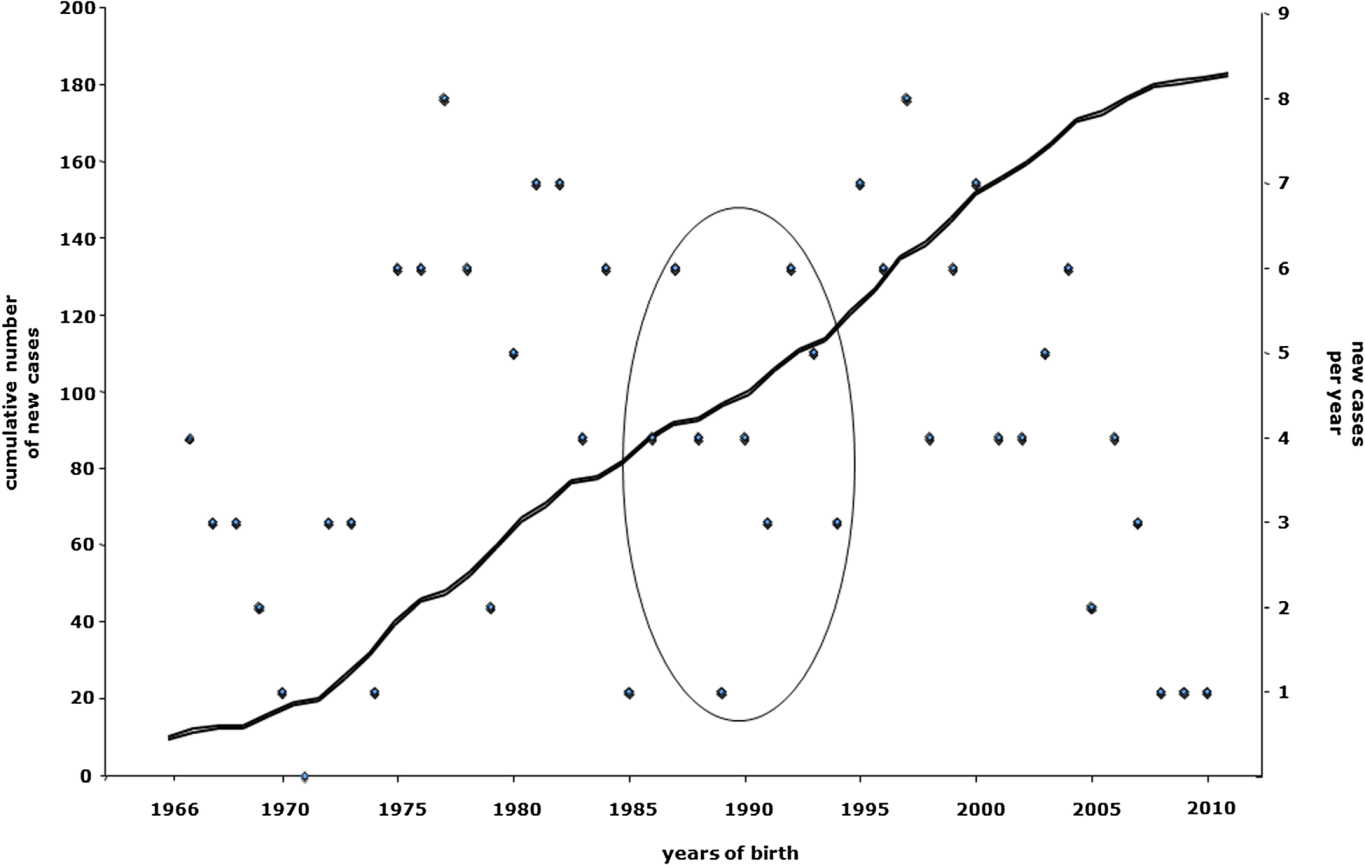
**Plotting of cumulative index of 183 NCL cases born in 1966–2010 as ascertained by CLNet.** Patients are sorted by year of birth and plotted by number of new cases per year (dark dots) and cumulative number of cases. The curve shows relatively steady recruitment of new cases over time, with a slight deflection from the mid-1980s to the mid-1990’s (circled). The flat ends of the curve are possibly related to under-diagnosis during the late-1960s to early-1970s, and the presence of still undiagnosed cases today (right end of the curve).

In mid-January 2005, 72 out of 183 NCL patients were still alive, giving a prevalence rate of 1.2/1,000,000. Plotting the cumulative index of 183 patients against year of birth resulted in a double S-shaped curve; the curve showed a slight deflection corresponding to lower annual occurrence of NCL cases over one decade (late 1980s to late 1990s). The flattened curve corresponding to the most recent years may be related to cases reported to CLNet Units but still awaiting diagnosis (Figure 1).

### Molecular data analysis

A total of 77 mutations were detected after examining 8 genes (see Additional file 1). Of the 112 children with molecular confirmation, 17 carried mutations in *CLN1*, in association with three phenotypes: INCL (n= 6), LINCL (n= 9) and JNCL (n= 2). Eleven different mutations were identified; four missense and seven predicting either truncated or severely affected protein. Five of the children with INCL carried either homozygous or compound heterozygous severe mutations, with 9/10 alleles predicting early protein truncation. The other harboured a homozygous missense mutation (c.722C>T/p.S241L), which might predict a less severe clinical course, notwithstanding the early onset. Of the nine patients with LINCL, six carried homozygous missense mutations (c.665 T>C/p.L222P being the most frequent), two were homozygous for an insertion leading to aberrant splicing, and the last was compound heterozygous for two nonsense variants [28]. Interestingly, the 3.6 Kb large deletion c.124+1214_235-102del was associated with a severe infantile form (with fatal outcome at age 6 years), when allelic to a nonsense mutation, whereas it gave origin to a less severe phenotype (still alive at 16 years) when in combination with a nucleotide change leading to a missense mutation. Two sibs, heterozygous for c.363-3 T>C, which predicts aberrant splicing, and c.541 G>A/p.V181M, showed a JNCL phenotype. The c.665 T>G/p.L222P mutation was the most frequent in the CLN1 patients, being detected in 29.4% of alleles.

The patients with mutations in *CLN2* were clinically homogeneous, all 29 presenting manifestations consistent with a LINCL phenotype. Seventeen different mutations were detected. Six were missense mutations predicting aminoacid changes. Five mutations were frameshifts or splice site variants, and six were nonsense mutations. The c.622C>T/p.R208* nonsense variant accounted for 20% of *CLN2* alleles. Altogether, molecular investigations of *CLN2* predicted a severely altered gene product in 65.5% of cases.

The classical juvenile phenotype was present in all the patients mutated in *CLN3*. Twelve patients carried the homozygous 1.02 Kb deletion, whereas one was a heterozygous compound. Two different homozygous nonsense mutations were detected in the remaining three patients. It is worth noting that none of the children of non-Italian ancestry was mutated in *CLN3*.

Forty-six LINCL children were found to have mutations in the *CLN5*-*CLN6*-*CLN7*-*CLN8* genes. A high rate of different mutations (as compared to number of alleles affected) was seen in each form, consistent with a marked genetic heterogeneity. Missense and nonsense mutations were evenly distributed in all the forms, except for *CLN8*, which showed a prevalence of missense mutations. In *CLN5* the c.772 T>G/p.Y258D mutation accounted for 1/3 of the alleles. Seventeen different disease-related variants were detected in the 21 patients carrying mutations in *CLN6*. Eleven were nucleotide changes that predicted an amino acid substitution, while three were frameshift and three nonsense mutations. Severe protein alterations were predicted in 10 cases. The mutations were evenly distributed, the most frequent being c.896C>T/p.P299L and c.662A>G/p.Y221C, together affecting a total of 10 alleles. No genotype-phenotype correlation could be drawn for this form. In *CLN7*, eight out of the 14 mutations were either splice site/frameshift variants or nonsense changes leading to an early stop codon. Severe protein alterations were predicted in 79% of cases. Mutations in *CLN8* were detected in seven patients: five nucleotide substitutions and two deletions. Finally, a single missense in *CLN10* was detected in one child. Once again, the genotype did not seem to predict the phenotype.

Findings showing the genetic heterogeneity of the cohort are summarized in Table 3.

**Table 3:** Genetic heterogeneity expressed as the number of mutations per examined gene in 124 NCL patients

**GENE**	**Patients**	**Alleles**	**Mutations**
***CLN1***	**17**	**34**	**11**
***CLN2***	**29**	**56**	**17**
***CLN3***	**16**	**32**	**3**
***CLN5***	**7**	**14**	**7**
***CLN6***	**21**	**42**	**17**
***CLN7***	**14**	**28**	**14**
***CLN8***	**7**	**14**	**7**
***CLN10***	**1**	**2**	**1**

## Discussion

In this study, we investigated the relative incidence of childhood forms of NCL in Italy and the phenotypic spectrum related to mutations in known NCL genes. Descriptive epidemiology data, collected through the application of shared clinical and morphological diagnostic criteria and direct gene sequencing of eight known NCL genes, revealed an incidence rate of 0.98/100,000 live births in the period 1992–2004. This figure is 58% higher than that recorded in the pre-genetic era [8], and the increase is probably due to the availability of new diagnostic procedures, such as biochemical and molecular testing, and greater awareness of the disease. The number of new NCL cases has been increasing constantly since the mid-1970s (Figure 1), as shown by steady rise in the cumulative index, which predicts about five new cases a year. Even though the years included in our incidence analysis belong to the molecular era, the incidence rate of NCLs in Italy was still found to be lower than in other European countries where epidemiological investigations were recently performed [16,29]. It is unclear whether this is due to an ascertainment bias, since our study might have missed cases presenting in late childhood/adolescence and not referred to neuropediatric centers (e.g., JNCL patients with visual problems or psychiatric manifestations alone might have escaped our registry survey) or to misdiagnosed cases. Furthermore, we may have missed a small number of cases diagnosed elsewhere before molecular analysis tools became available in Italy. Indeed, between the late 1980s and the late 1990s there appeared to be fewer children born with NCL (Figure 1), and this may be because some Italian patients recorded in the NCL mutation database [6] or reported in the literature [25,30,31] were not subsequently included in the CLNet database. This discrepancy might also be explained by the still limited proportion (16%) of cases, in the CLNet database, occurring in non-Italian ethnicities. In this regard, Italy differs from other European countries, such as United Kingdom, the Netherlands, and Germany, where higher levels of immigration from the Middle East, Asia and countries with high rates of inbreeding result in a much broader ethic mix of NCL patients.

All the known NCL forms and variants represented in the CLNet database were evenly distributed (see Table 2). LINCL appear to be the most frequent forms, accounting for an incidence of 0.78/100,000 live births. About half of the cohort had a genetic diagnosis of LINCL, the single most affected gene being *CLN2*, found to be mutated in 23.5% of the entire sample. High frequencies of LINCL and *CLN2* mutations have been reported in only two previous studies, carried out in restricted geographic areas, namely British Columbia [32] and Newfoundland [15], respectively. In the latter study, LINCL patients mutated in *CLN5* and *CLN6* accounted for 26% of the whole LINCL population.

Nearly 17% of patients of this cohort were mutated in *CLN6*. Two phenotypes were identified: a subtype characterized by late infantile onset and survival into the second decade, and a late juvenile/adolescent form with a disease course protracted into adulthood. Three clinical variants of *CLN1* mutations were identified, and phenotypic expression was apparently unrelated to the kind of mutation (and the predicted effects on the gene product). Relatively few children in our sample were affected by INCL, a result which is at odds with the pattern in countries such as Finland where a founder effect has been recognized [33]. The frequency of *CLN3*-mutated patients was also relatively low compared with the worldwide incidence rates, and also considering the allegedly frequent occurrence of mutations in *CLN3* in Northern Europe. The apparent discrepancy in our study between the numbers of clinical diagnoses and of confirmatory molecular tests (see Table 1 and Table 2) might be due to the fact that before testing was possible in our country all genetic analyses were made abroad. The distribution of Italian JNCL cases by year of birth differs from that other NCL forms, failing to show significant differences between the pre- and post-molecular era. Conversely, there was a surge in LINCL diagnoses once molecular testing became available. Prospective studies will probably better indicate the rate of relative frequencies for juvenile NCL forms.

As expected, given the heterogeneity of the clinical spectrum of childhood NCL as a whole, we did not observe clear-cut phenotype-genotype relationships. However, in INCL and LINCL secondary to mutated *CLN2* — the most severe forms in terms of signs/symptoms at onset, disease progression and survival — the majority of mutations predicted severe loss of function. Similarly, *CLN7* showed a high frequency of mutations expected to result in markedly abnormal gene product expression (78.6%). Accordingly, a mutation in *CLN7* was a bad predictor of clinical severity among the LINCL cases in our cohort. Only one child in this cohort harbored a *CLN10* mutation, underlining the rarity of this condition [34].

As shown elsewhere [35-37], about 10% of the NCL cases in this survey remained without a genetic diagnosis. Therefore, new NCL genes need to be identified, and the availability of informative families will help in accomplishing this. However, we cannot rule out that some of the CLNX patients of this cohort might display mutations in either *ATP13A2* or *KCTD7*, even if their phenotypes are markedly different form the reported cases [26,27].

With the exception of the CLN3 form, NCL in Italy seems to be characterized by heterogeneity, both clinical (phenotypes) and genetic (number of mutations/genes). The finding of a high number of private mutations without geographic clusters and the lack of large families with multiple affected children suggests that Italian NCL cases have an assorted genetic background. This variability might reflect the fact that Italy, a country lying on migratory routes and with a relatively low rate of inbreeding, has historically known several different dominations, and frequent mixing between the local population and invaders.

The enhanced knowledge of the epidemiology of NCL in Italy, particularly the figures of prevalence and the prediction of new cases per year, together with increase knowledge of the long term complications of the disease(s) should help a better allocation of health resources for planning sufficient care in children and their families. It will also help to increase the awareness about this group of diseases, possibly shortening the time lag between onset and the definite diagnosis.

## Conclusions

In the period 1992–2004 the incidence of NCLs in Italy was 0.98/100,000 live births, a rate higher than would have been expected in the “pre-DNA” era but relatively lower than what is observed in other European countries. With Italy now seeing increasing rates of immigration of highly consanguineous populations from North African countries and Eastern Europe, it is tempting to hypothesize that these figures will change again in future investigations.

## Abbreviations

NCL: Neuronal Ceroid Lipofuscinosis; CLNet: Ceroid Lipofuscinosis collaborative Network; LINCL: Late Infantile Neuronal Ceroid Lipofuscinosis; JNCL: Juvenile Neuronal Ceroid Lipofuscinosis.

## Competing interests

The authors declare that they have no competing interest.

## Authors’ contributions

FMS, BG, MF and FP performed molecular and biochemical analyses, collected data from earlier studies, and revised the draft of the manuscript. FC retrieved epidemiological data from a previous study. NN, BDB, SS, ASu, RBi, RBa collected clinical data of most NCL patients. AS designed the study, drafted the manuscript, and revised the last version of the paper. All authors read and approved the final manuscript.

## Supplementary Material

Additional file 1 **Supplementary Material Legend.** List of mutations detected after screening 124 patients for NCL genes. Mutations not listed in the NCL mutation database [http://www.ucl.ac.uk/ncl/mutation] are in bold. Click here for file
